# Blood Biomarkers Predict Survival Outcomes in Patients with Hepatitis B Virus-Induced Hepatocellular Carcinoma Treated with PD-1 Inhibitors

**DOI:** 10.1155/2022/3781109

**Published:** 2022-08-17

**Authors:** Rui Huang, Yanfen Zheng, Wenxue Zou, Chao Liu, Jibing Liu, Jinbo Yue

**Affiliations:** ^1^Department of Graduate, Shandong Cancer Hospital and Institute, Shandong First Medical, University and Shandong Academy of Medical Sciences, Jinan, Shandong, China; ^2^Department of Radiation Oncology, Shandong Cancer Hospital and Institute, Shandong, First Medical University and Shandong Academy of Medical Sciences, Jinan Shandong, China; ^3^Department of Interventional Radiology, Shandong Cancer Hospital and Institute, Shandong First Medical University and Shandong Academy of Medical Sciences, Jinan, Shandong, China

## Abstract

**Purpose:**

To investigate the prognostic value of blood markers in patients with hepatitis B virus (HBV)-induced hepatocellular carcinoma (HCC) treated with PD-1 inhibitors. *Patients and Methods*. We retrospectively collected and analyzed the clinicopathological data of 110 HBV-induced HCC patients treated with PD-1 inhibitors. Progression-free survival (PFS) and overall survival (OS) were scrutinized using Kaplan-Meier analysis and the log-rank test, and all potential risk factors were analyzed with univariate and multivariate Cox regression analyses.

**Results:**

The mean OS and PFS were 6.5 and 5.5 months, respectively. According to Kaplan-Meier survival curves, elevated systemic immune-inflammation index (SII), platelet-to-lymphocyte ratio (PLR), and neutrophil-to-lymphocyte ratio (NLR) correlated with decreased OS and PFS (all *P* < 0.05), and low lymphocyte-to-monocyte ratio (LMR) correlated with decreased PFS and OS (all *P* < 0.05). Per multivariate Cox regression analyses, SII, PLR, and portal vein tumor thrombus (PVTT) correlated independently with PFS (all *P* < 0.05), whereas SII, PLR, NLR, and portal vein tumor thrombus (PVTT) correlated with OS (all *P* < 0.05).

**Conclusion:**

SII, PLR, and PVTT predicted OS and PFS in HCC patients who received PD-1 inhibitors and, therefore, could be useful predictors for risk stratification and individualized therapeutic decision-making for patients with HBV-induced HCC treated with PD-1 inhibitors.

## 1. Introduction

Hepatocellular carcinoma (HCC) accounts for 75-85% of primary liver cancers and is the sixth most common cancer worldwide [1]. More than half of the new cases diagnosed in the world are from China [2]. HCC is caused primarily by hepatitis B virus (HBV) or hepatitis C virus (HCV) infections, autoimmune hepatitis, alcoholic hepatitis, and other metabolic diseases. Its main treatment modalities include surgery, transarterial chemoembolization (TACE), liver transplantation, radiofrequency ablation, radiotherapy (RT), targeted agents, and systemic chemotherapy; a comprehensive treatment model based on surgery has been formed gradually. Nevertheless, advanced HCC patients do not respond well to these treatment modalities.

In recent years, a series of breakthroughs in tumor immunotherapy has seen a variety of immune checkpoint inhibitors (ICIs) emerge as suitable treatment options for advanced HCC patients; these have demonstrated not only sustained responses in select groups of patients but also a better safety profile than targeted agents [3–5]. Given the success of ICIs, predictive biomarkers for patients who respond better to ICIs must be identified, particularly as PD-L1 expression in tumor cells does not correlate with the response demonstrated by HCC patients who receive PD-1 inhibitors.

Microsatellite instability (MSI), an outcome of deficient DNA mismatch repair (DMMR), is the first predictive biomarker for a PD-1 inhibitor approved by the United States Food and Drug Administration (FDA). MSI-H colon cancer has responded well to ICIs, but it appears to be a rare event in HCC. Recently, next-generation sequencing (NGS) has identified WNT/*β*-catenin mutations as potential biomarkers for predicting ICI resistance in advanced HCC patients; however, the high cost and complexity of NGS has limited its clinical application widely [6]. Various other predictive biomarkers have been reported, including des-carboxy prothrombin (DCP), vascular endothelial growth factor (VEGF), hepatic growth factor (HGF), osteopontin (OPN), angiopoietin-2 (Ang-2), Glypican-3, c-MET, Golgi protein 73 (Gp-73), and insulin-like growth factor-1 (IGF-1) [7]. Yet, none of these aforementioned or others have been recognized or validated in HCC. Hence, to identify patients who may benefit from anti-PD-1 therapy, it is imperative to seek and develop predictive biomarkers that are clinically and economically feasible.

Mounting evidence suggests that inflammation affects tumor initiation, proliferation, angiogenesis, metastasis, and response to chemotherapy and RT. As a result, several inflammatory markers consisting of inflammation and immune cell components have been postulated [8], including systemic immune-inflammation index (SII), platelet-to-lymphocyte ratio (PLR), neutrophil-to-lymphocyte ratio (NLR), and lymphocyte-to-monocyte ratio (LMR). High SII, PLR, NLR, and low LMR reflect either an increased platelet count (promotion of the exudation of tumor cells) [9], an increased neutrophil count (increase in the permeability of blood vessels, infiltration rate of tumor vessels, and seeding amount), or a reduced lymphocyte count (decrease in the ability of antitumor immunity). SII is associated with poor outcomes in some malignancies, including colorectal cancer, postoperative pulmonary neuroendocrine tumors (PNETs), soft tissue sarcomas, postoperative nonmetastatic renal cell carcinoma (RCC), breast cancer, postradical resection cervical cancer, upper segment urothelial cancer, and postradical resection hepatocellular carcinoma [10–16]. NLR predicts how patients with advanced melanoma respond to immunotherapy and the recurrence of glioma; it also serves as a prognostic indicator for malignancies, such as renal cell carcinoma, metastatic gallbladder cancer, prostate cancer, and pediatric mucoepidermoid carcinoma [17–22]. NLR and PLR are independent prognostic factors in patients with metastatic NSCLC treated with nivolumab, and they also predict gastric cancer, chemotherapy response and prognosis in patients with advanced gastric cancer, and pathological complete remission after neoadjuvant chemotherapy in breast cancer patients, as well as serve as an indicator of poor prognosis in resectable liver cancer [23–26]. LMR can be used as a prognostic biomarker for stage IV non-small-cell lung cancer (NSCLC), pancreatic neuroendocrine tumors, ovarian cancer, cervical cancer, breast cancer bone metastases, gallbladder cancer, follicular non-Hodgkin's lymphoma, and other malignancies [27–33].

In light of the differing roles of these inflammatory markers (SII, PLR, NLR, and LMR), we aimed to further clarify their prognostic significance in HCC after two cycles of treatment of the disease with PD-1 inhibitors.

## 2. Materials and Methods

### 2.1. Patient Selection

Patients were diagnosed with HCC based on findings with dynamic computed tomography and elevations in their *α*-fetoprotein levels, contrast-enhanced ultrasonography, magnetic resonance imaging, or pathology following the guidelines of the Chinese Diagnosis and Treatment of Primary Liver Cancer (2019 Edition). We retrospectively collected only the clinicopathological data of patients with advanced HCC whose first anti-PD-1 therapy occurred between February 2019 and September 2020 at the Shandong Cancer Hospital and Institute. Excluded from this study were patients with insufficient clinical data at the time of our research; patients with acute inflammation, autoimmune diseases, or hematological diseases or transplant history; and patients treated with antibiotics, hormones, or immunosuppressants during or one month prior to anti-PD-1 therapy. The ethics committee of Shandong Cancer Hospital and Institute has approved the present study, and each participant has signed a written informed consent.

### 2.2. Data Collection

The following characteristic clinical data were collected from the medical record system of patients: gender, age, smoking history, cancer stage, initial therapeutic response, Eastern Cooperative Oncology Group performance score, body mass index (BMI), treatment modality, blood biomarkers (including lymphocytes, neutrophils, platelets, *α*-fetoprotein, and monocytes), disease progression date, and last follow-up status. The HCC stage was assigned per the China Liver Cancer (CNLC) classification system and Barcelona Clinic Liver Cancer (BCLC) classification system [34].

### 2.3. Definitions

The blood samples of all patients were collected on days 1-7 before the primary treatment and the third cycle of treatment. SII was defined as platelet count × neutrophil count/lymphocyte count. We assessed the liver function using the Child-Pugh classification system. Overall survival (OS) was defined as the date from anti-PD-1 therapy to the date of death due to any cause or to the time of the last follow-up. Progression-free survival (PFS) was described as the time from anti-PD-1 therapy to the time of disease recurrence or metastasis.

### 2.4. Treatment of HCC

Based on the discussion of multidisciplinary teams (MDTs) in Shandong Cancer Hospital and Institute, patients' primary tumors were treated locally with radiofrequency ablation (RFA), transarterial chemoembolization (TACE), RT, or liver cancer resection, as well as first-line systemic therapy, including sorafenib or lenvatinib treatment combined with more than two cycles of anti-PD-1 therapy.

### 2.5. Statistical Analysis

Chi-squared analysis and student's t-test were used to compare categorical and continuous variables between two groups. Receiver operator characteristic (ROC) curves were constructed to calculate the area under the curve (AUC) and determine statistically significant variables. The maximum values of the Youden indexes of SII, PLR, NLR, and LMR were determined using the ROC curve analysis, and the corresponding expression levels of the maximum values were the truncated values of SII, PLR, NLR, and LMR. OS and PFS were analyzed using the Kaplan-Meier method, and intergroup comparisons of survival data were calculated using the log-rank test. Factors significantly associated with the OS and PFS in univariate analyses were used as covariates for multivariate Cox proportional hazards models, with variables with *P* values <0.2 in univariate analyses selected for multivariate analyses. A *P* value of <0.05 was considered statistically significant. All analyses were performed using the SPSS software version 23.

## 3. Results

### 3.1. Patient Characteristics

**Figure 1:**
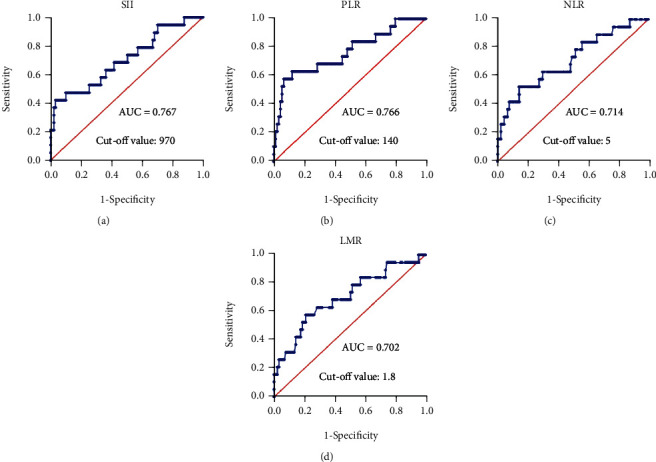
Receiver operating characteristic curves were generated to evaluate the discriminatory ability of the systemic immune-inflammation index, platelet-to-lymphocyte ratio, neutrophil-to-lymphocyte ratio, and lymphocyte-to-monocyte ratio. SII: systemic immune-inflammation index; PLR: platelet-to-lymphocyte ratio; NLR: neutrophil-to-lymphocyte ratio; LMR: lymphocyte-to-monocyte ratio.

We selected blood samples before the third cycle of treatment to explore the relationship between inflammatory markers (SII, PLR, NLR, and LMR) and survival outcomes in HBV-induced HCC patients receiving anti-PD-1 therapy. Patient characteristics are outlined in Table 1. The median age was 54.5 years (range 31-84), with a predominantly male population (90.9%). Ninety-eight patients (89.1%) had Child-Pugh grade A liver function, 110 (100%) had hepatitis B virus (HBV), and 92 (83.6%) were at the BCLC stage of C versus 18 (16.4%) at the stage of B. The AUC values for SII, PLR, NLR, and LMR that predicted survival status in HCC patients were 0.707, 0.766, 0.712, and 0.702, respectively (95% CI: 0.568-0.846, *P* = 0.0047; 95% CI: 0.635-0.898, *P* = 0.0003; 95% CI: 0.579-0.849, *P* = 0.0035; 95% CI: 0.565-0.839, *P* = 0.0058; , respectively, Figure 1). The corresponding selected cutoff values for SII, PLR, NLR, and LMR were 5, 140, 970, and 1.8.

### 3.2. Progression-Free Survival

**Figure 2:**
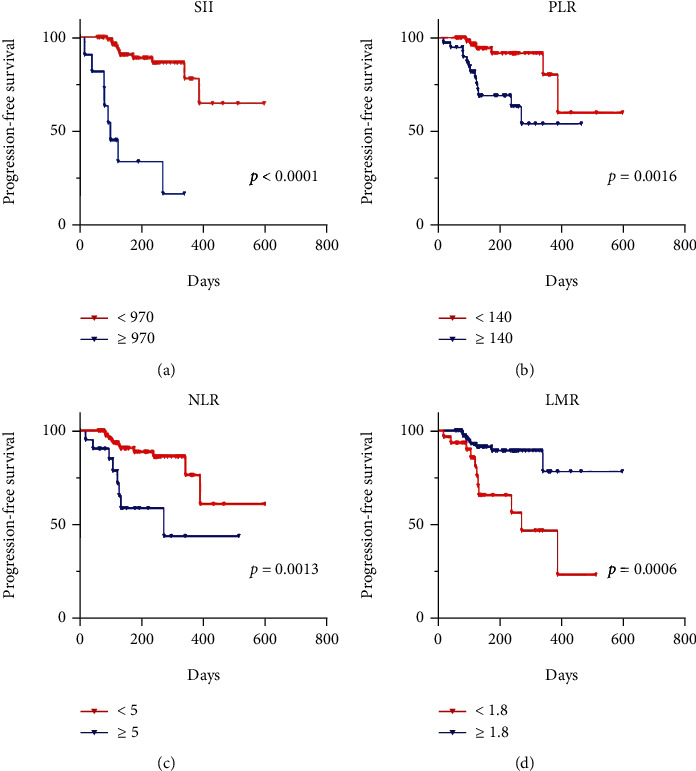
The Kaplan–Meier survival curves indicated that elevated SII, PLR, and NLR were associated with decreased PFS (a–c). Conversely, decreased LMR was associated with decreased PFS (d).

The median time for tumor progression was 5.5 months (95% CI: 0.6-20 months). According to Kaplan-Meier survival curves, elevated SII, PLR, and NLR were associated with decreased PFS in HCC patients (6.7 vs. 4.5 months, *P* < 0.0001; 6.7 vs. 5.9 months, *P* = 0.0016; 6.7 vs. 5.4 months, *P* = 0.0013, respectively), but lower LMR was linked to lower PFS (6.8 vs. 5.5 months, *P* = 0.0006; Figure 2).

Per univariate Cox regression analysis, SII, PLR, NLR, and LMR were significant predictors of PFS after the administration of anti-PD-1 s to HCC patients; the China Liver Cancer (CNLC) stage and portal vein tumor thrombus (PVTT) were also statistically significant (Table 2). Multivariate Cox regression analyses revealed that SII (HR, 7.112; 95% CI: 2.373-21.314; *P* < 0.0001), PLR (HR, 4.995; 95% CI: 1.614-15.463; *P* = 0.005), and PVTT (*P* < 0.05) were significant predictors of PFS in patients with HBV-induced HCC (Tables 3 and 4, Supplementary Table 1-2).

### 3.3. Overall Survival

**Figure 3:**
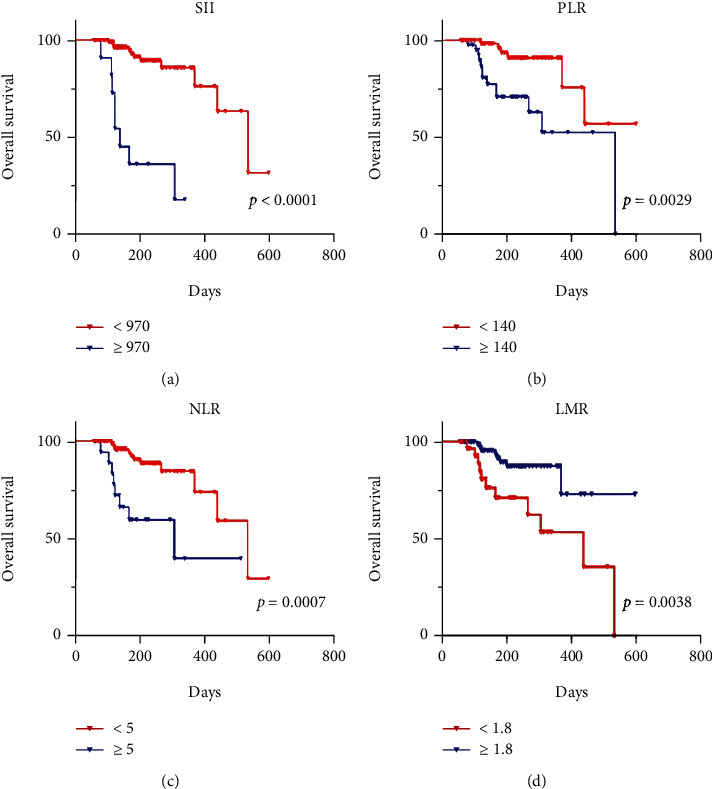
The Kaplan–Meier survival curves indicated that elevated SII, PLR, and NLR were associated with decreased OS (a–c). Conversely, decreased LMR was associated with decreased OS (d).

At the time of analysis, 91 (82.7%) patients remained alive. The median survival was 6.4 months (95% CI: 2.0-20). According to Kaplan-Meier survival curves, elevated SII, PLR, and NLR were associated with reduced OS in HCC patients (7.2 vs. 5.9 months, *P* < 0.0001; 7.3 vs. 6.7 months, *P* = 0.0029; 7.3 vs. 6.0 months, *P* = 0.0007), but low LMR was linked to low OS (7.2 vs. 6.7 months, *P* = 0.0038; Figure 3).

Per univariate Cox regression analysis, SII, PLR, NLR, and LMR were significant predictors of OS after the administration of anti-PD-1s to HCC patients; the PVTT was also statistically significant (Table 5). Multivariate Cox regression analyses revealed that SII (HR, 5.564; 95% CI: 1.781-17.381; *P* = 0.003), PLR (HR, 3.002; 95% CI: 1.070-8.421; *P* = 0.037), NLR (HR, 3.398; 95% CI: 1.050-10.995; *P* = 0.041), and PVTT (*P* < 0.05) were significant predictors of OS in patients with HBV-induced HCC (Tables 6–8, Supplementary Table 3).

## 4. Discussion

In recent years, many studies have explored the impact of inflammatory markers on patients with hepatocellular carcinoma receiving different treatment modalities. For example, preoperative NLR and PLR are proven indicators of poor prognosis in resectable hepatocellular carcinoma, NLR and PLR are predictors of tumor response after drug-eluting bead transarterial chemoembolization for hepatocellular carcinoma, and SII is indicative of patient prognosis after radical resection for hepatocellular carcinoma [16, 35, 36]. Our retrospective research reached similar conclusions. However, we demonstrated a more comprehensive impact of inflammatory markers on HCC patients: (1) The AUC values of SII, PLR, NLR, and LMR were 0.717, 0.766, 0.712, and 0.702, respectively, suggesting that these biomarkers are feasible predictors of the survival outcome of HBV-induced HCC patients receiving anti-PD-1 therapy; (2) SII and PLR were valuable predictive biomarkers of PFS in HBV-induced HCC patients receiving anti-PD-1 drugs; (3) patients with high SII, PLR, or NLR had shorter OS compared to their counterparts with lower SII, PLR, and NLR; (4) PVTT could also predict OS and PFS in HCC patients who received PD-1 inhibitors.

Several published studies have shown how critical inflammation is to cancer progression [37–39]. HCC is a typical inflammatory disease caused primarily by underlying cirrhosis, fibrosis, and chronic liver inflammation. Inflammation promotes HCC progression by recruiting proinflammatory cytokines and regulatory T lymphocytes, activating downstream STAT3 and NF-*κ*B, and inducing a decline in immune cell and inflammatory cell production, leading to an imbalanced host immune response and subsequently to the loss of response to chemotherapy [40–42]. As a result, researchers and clinicians have shown particular interest in the link between inflammation and the prognosis or occurrence of liver cancer over the past few decades.

Recently, an investigation revealed that the platelet-to-lymphocyte and neutrophil-to-lymphocyte ratios are predictive of HCC in patients treated with PD-1 inhibitors [43]. Compared to that investigation, our research has the following advantages: (1) The HBV infection rate in our study was 100%, whereas the HBV infection rate in Dharmapuri et al.'s investigation was 32%; therefore, our research was more reliable in predicting the survival outcome of patients with HBV-induced HCC. (2) Our research excluded patients with acute inflammation and those treated with antibiotics, hormones, and immunosuppressants during or one month prior to anti-PD-1 therapy, for these drugs and acute inflammation affect lymphocyte and neutrophil counts, whereas Dharmapuri et al. did not exclude this group. (3) We also analyzed SII and LMR and found that LMR correlated positively with the prognosis of HCC patients, while SII correlated negatively. Our study is consistent with that of Dharmapuri et al. in the finding that low NLR and PLR are independent predictors of improved survival; NLR and PLR values seem to be better predictors of survival differences after treatment than pretreatment.

Despite its revelations, our study has several limitations. First, it was a retrospective study, and it was impractical to control for consistency in the way patients were treated prior to enrollment. Second, there are no well-defined cutoff values for SII, PLR, and NLR; our results for the cutoffs for these inflammatory markers must, hence, be verified or redefined in the verification queue. Finally, lymphocyte subsets were not measured; consequently, the specific underlying mechanisms must be investigated further.

## 5. Conclusion

SII, PLR, and PVTT were significant predictors of PFS and OS; NLR could also predict OS. Therefore, SII and PLR are typical inflammatory markers that predict PFS and OS in HBV-induced HCC patients receiving anti-PD-1 therapy and could be useful biomarkers for risk stratification and treatment decisions for HCC patients.

## Figures and Tables

**Table 1:** Patient characteristic.

Variable	Number of patients	%
Patient number	110	100
Gender		
Male	100	90.9
Female	10	9.1
Age (year) median (range)	54.5 (31-84)	
ECOG performance score		
0-1	78	70.9
≥2	32	29.1
Child-Pugh class		
A	98	89.1
B	12	10.9
BMI		
<25	80	72.7
≥25	30	27.3
Viral hepatitis		
HBV	110	100
BCLC stage		
B	18	16.4
C	92	83.6
CNLC stage		
IIb	18	16.4
IIIa	23	20.9
IIIb	69	62.7
Treatment of primary tumor		
TACE	87	79.1
RFA	15	13.6
RT	25	22,7
Resection of liver cancer	33	30
None	15	13.6
The cycles of anti-PD-1 therapy		
Two cycles	55	50.0
More than three cycles	55	50.0
PVTT		
Yes	65	59.1
None	45	40.9
AFP level		
<400 ng/ml	49	44.5
≥400 ng/ml	61	55.5
Lymphocyte ×10^9/LMedian (range)	1.233 (0.18-4.09)	
Neutrophil ×10^9/L median (range)	3.339 (0.97-10.53)	
Monocyte ×10^9/L median (range)	0.46 (0.13-1.38)	
Platelet ×10^9/L median (range)	141.67 (27-496)	
SII		
<970	99	90.0
≥970	11	10.0
PLR		
<140	69	62.7
≥140	41	37.3
NLR		
<5	89	80.9
≥5	21	19.1
LMR		
<1.8	31	28.2
≥1.8	79	71.8

**Table 2:** Univariate Cox proportional hazards regression models for progression-free survival.

Variable	Univariate analysis		
	HR	95% CI	*P* value
Gender			
Male vs. female	1.427	0.52-3.915	0.49
Age			
<60 vs. ≥60	1.230	0.454-3.334	0.684
ECOG performance score			
≥2 vs. <2	1.733	0.695-4.319	0.238
BMI			
<25 vs. ≥25	1.559	0.548-4.440	0.405
Child-Pugh class			
B vs. A	2.152	0.697-6.643	0.183
BCLC stage			
C vs. B	2.655	0.351-20.097	0.344
CNLC stage			
IIIb vs. IIb and IIIa	4.992	1.152-21.631	0.032
PVTT			
Yes vs. none	14.290	1.907-107.102	0.010
AFP			
≥400 vs. <400 ng/ml	2.394	0.895-8.400	0.082
SII			
≥970 vs. <970	12.72	4.863-33.271	< 0.0001
PLR			
≥140vs. <140	4.211	1.594-11.126	0.004
NLR			
≥5 vs. <5	3.990	1.599-9.959	0.003
LMR			
≥1.8 vs. <1.8	0.229	0.092-0.572	0.002

**Table 3:** Multivariate Cox proportional hazards regression models for progression-free survival.

Variable	Multivariate analysis		
	HR	95% CI	*P* value
Child-Pugh class			
B vs. A	0.799	0.185-3.453	0.764
CNLC stage			
IIIb vs. IIb and IIIa	3.986	0.803-19.774	0.901
AFP			
≥400 vs. <400 ng/ml	1.935	0.697-5.376	0.205
PVTT			
Yes vs. none	8.189	1.027-65.288	0.047
SII			
≥970 vs. <970	7.112	2.373-21.314	<0.0001

**Table 4:** Multivariate Cox proportional hazards regression models for progression-free survival.

Variable	Multivariate analysis		
	HR	95% CI	*P* value
Child-Pugh class			
B vs. A	0.963	0.236-3.935	0.958
CNLC stage			
IIIb vs. IIb and IIIa	3.337	0.706-15.759	0.128
AFP			
≥400 vs. <400 ng/ml	1.462	0.516-4.138	0.475
PVTT			
Yes vs. none	17.972	2.216-145.779	0.007
PLR			
≥140vs. <140	4.995	1.614-15.463	0.005

**Table 5:** Univariate Cox proportional hazards regression models for overall survival.

Variable	Univariate analysis		
	HR	95% CI	*P* value
Gender			
Male vs. female	1.377	0.500-3.791	0.536
Age			
<60 vs. ≥60	1.056	0.392-2.843	0.915
ECOG performance score			
≥2 vs. <2	1.830	0.721-4.646	0.203
BMI			
<25 vs. ≥25	1.589	0.565-4.473	0.380
Child-Pugh class			
B vs. A	2.162	0.694-6.738	0.184
BCLC stage			
C vs. B	2.164	0.284-16.466	0.456
CNLC stage			
IIIb vs. IIb and IIIa	4.088	0.938-17.805	0.061
PVTT			
Yes vs. none	14.110	1.882-105.773	0.010
AFP			
≥400 vs. <400 ng/ml	2.114	0.801-5.580	0.131
SII			
≥970 vs. <970	10.638	3.972-28.492	<0.0001
PLR			
≥140vs. <140	3.910	1.483-10.314	0.006
NLR			
≥5 vs. <5	4.388	1.726-11.158	0.002
LMR			
≥1.8 vs. <1.8	0.278	0.110-0.700	0.007

**Table 6:** Multivariate Cox proportional hazards regression models for overall survival.

Variable	Multivariate analysis		
	HR	95% CI	*P* value
Child-Pugh class			
B vs. A	0.746	0.185-3.014	0.681
CNLC stage			
IIIb vs. IIb and IIIa	2.910	0.599-14.129	0.185
AFP			
≥400 vs. <400 ng/ml	1.708	0.602-4.847	0.314
PVTT			
Yes vs. none	8.220	1.027-65.788	0.047
SII			
≥970 vs. <970	5.564	1.781-17.381	0.003

**Table 7:** Multivariate Cox proportional hazards regression models for overall survival.

Variable	Multivariate analysis		
	HR	95% CI	*P* value
Child-Pugh class			
B vs. A	1.167	0.314-4.341	0.818
CNLC stage			
IIIb vs. IIb and IIIa	2.801	0.593-13.238	0.194
AFP			
≥400 vs. <400 ng/ml	1.207	0.426-3.423	0.723
PVTT			
Yes vs. none	12.068	1.551-93.910	0.017
PLR			
≥140vs. <140	3.002	1.070-8.421	0.037

**Table 8:** Multivariate Cox proportional hazards regression models for overall survival.

Variable	Multivariate analysis		
	HR	95% CI	*P* value
Child-Pugh class			
B vs. A	0.693	0.168-2.861	0.612
CNLC stage			
IIIb vs. IIb and IIIa	3.727	0.778-17.860	0.100
AFP			
≥400 vs. <400 ng/ml	1.830	0.617-5.431	0.276
PVTT			
Yes vs. none	8.943	1.129-70.813	0.038
NLR			
≥5 vs. <5	3.398	1.050-10.995	0.041

Abbreviations: ECOG: Eastern Cooperative Oncology Group; HBV: hepatitis B virus; BMI: body mass index; BCLC: Barcelona Clinic Liver Cancer; CNLC: China Liver Cancer staging; RFA: radiofrequency ablation; TACE: transarterial chemoembolization; RT: radiotherapy; PVTT: portal vein tumor thrombus; AFP: *α*-fetoprotein; SII: systemic immune-inflammation index; PLR: platelet-to-lymphocyte ratio; NLR: neutrophil-to-lymphocyte ratio; LMR: lymphocyte-to-monocyte ratio.

## Data Availability

The data that support the findings of this study are available from the corresponding author upon reasonable request.

## Supplementary Materials

Supplementary Materials Supplementary Table 1: multivariate Cox proportional hazards regression models for progression-free survival. Supplementary Table 2: multivariate Cox proportional hazards regression models for progression-free survival. Supplementary Table 3: multivariate Cox proportional hazards regression models for overall survival. Supplementary Table 4: Wilcoxon signed-rank test of blood markers before and after treatment.

## References

[1] Bray F., Ferlay J., Soerjomataram I., Siegel R. L., Torre L. A., Jemal A. (2018). Global cancer statistics 2018: GLOBOCAN estimates of incidence and mortality worldwide for 36 cancers in 185 countries. *CA: a Cancer Journal for Clinicians*.

[2] McGlynn K. A., Petrick J. L., London W. T. (2015). Global epidemiology of hepatocellular carcinoma: an emphasis on demographic and regional variability. *Clinics in Liver Disease*.

[3] Zaragoza J., Caille A., Beneton N. (2016). High neutrophil to lymphocyte ratio measured before starting ipilimumab treatment is associated with reduced overall survival in patients with melanoma. *The British Journal of Dermatology*.

[4] Llovet J. M., Ricci S., Mazzaferro V. (2008). Sorafenib in advanced hepatocellular carcinoma. *The New England Journal of Medicine*.

[5] Finn R. S., Ryoo B. Y., Merle P. (2020). Pembrolizumab as second-line therapy in patients with advanced hepatocellular carcinoma in KEYNOTE-240: a randomized, double-blind, phase III trial. *Journal of Clinical Oncology*.

[6] Harding J. J., Nandakumar S., Armenia J. (2019). Prospective genotyping of hepatocellular carcinoma: clinical implications of next-generation sequencing for matching patients to targeted and immune therapies. *Clinical Cancer Research*.

[7] Piñero F., Dirchwolf M., Pessôa M. G. (2020). Biomarkers in hepatocellular carcinoma: diagnosis, prognosis and treatment response assessment. *Cells*.

[8] Dolan R. D., Lim J., McSorley S. T., Horgan P. G., McMillan D. C. (2017). The role of the systemic inflammatory response in predicting outcomes in patients with operable cancer: systematic review and meta-analysis. *Scientific Reports*.

[9] Labelle M., Begum S., Hynes R. O. (2011). Direct signaling between platelets and cancer cells induces an epithelial-mesenchymal-like transition and promotes metastasis. *Cancer Cell*.

[10] Dong M., Shi Y., Yang J. (2020). Prognostic and clinicopathological significance of systemic immune-inflammation index in colorectal cancer: a meta-analysis. *Therapeutic Advances in Medical Oncology*.

[11] Hou T., Guo T., Nie R. (2020). The prognostic role of the preoperative systemic immune-inflammation index and high-sensitivity modified Glasgow prognostic score in patients after radical operation for soft tissue sarcoma. *European Journal of Surgical Oncology*.

[12] Hu X., Shao Y. X., Yang Z. Q., Dou W. C., Xiong S. C., Li X. (2020). Preoperative systemic immune-inflammation index predicts prognosis of patients with non-metastatic renal cell carcinoma: a propensity score-matched analysis. *Cancer Cell International*.

[13] Zhang Y., Sun Y., Zhang Q. (2020). Prognostic value of the systemic immune-inflammation index in patients with breast cancer: a meta-analysis. *Cancer Cell International*.

[14] Zheng Y., Yu D., Yu Z. (2020). Association of preoperative systemic immune-inflammation index and prognostic nutritional index with survival in patients with upper tract urothelial carcinoma. *Journal of Cancer*.

[15] Huang H., Liu Q., Zhu L. (2019). Prognostic value of preoperative systemic immune-inflammation index in patients with cervical cancer. *Scientific Reports*.

[16] Hu B., Yang X. R., Xu Y. (2014). Systemic immune-inflammation index predicts prognosis of patients after curative resection for hepatocellular carcinoma. *Clinical Cancer Research*.

[17] Ma L., Li G., Wei M. (2020). Neutrophil-to-lymphocyte ratio and its changes are related to grade II-IV glioma recurrence. *Cancer Management and Research*.

[18] Pedersen M. M., Donskov F., Pedersen L., Zhang Z. F., Nørgaard M. (2020). Elevated neutrophil-lymphocyte ratio combined with hyponatremia indicate poor prognosis in renal cell carcinoma. *Acta Oncologica*.

[19] Wang Y., Dong X., Qu Z., Peng K., Sun X., Chen R. (2020). Correlation between peripheral blood neutrophil-lymphocyte ratio and CD34 expression in prostate cancer. *BMC Cancer*.

[20] Capone M., Giannarelli D., Mallardo D. (2018). Baseline neutrophil-to-lymphocyte ratio (NLR) and derived NLR could predict overall survival in patients with advanced melanoma treated with nivolumab. *Journal for Immunotherapy of Cancer*.

[21] Gao H., Gao Q., Sun J. (2020). Significance of pretreatment neutrophil-to-lymphocyte ratio in mucoepidermoid carcinoma of pediatrics: a multicenter study. *Frontiers in pediatrics.*.

[22] Mady M., Prasai K., Tella S. H. (2020). Neutrophil to lymphocyte ratio as a prognostic marker in metastatic gallbladder cancer. *HPB: the official journal of the International Hepato Pancreato Biliary Association.*.

[23] Fang T., Wang Y., Yin X. (2020). Diagnostic sensitivity of NLR and PLR in early diagnosis of gastric cancer. *Journal of Immunology Research*.

[24] Graziano V., Grassadonia A., Iezzi L. (2019). Combination of peripheral neutrophil-to-lymphocyte ratio and platelet-to-lymphocyte ratio is predictive of pathological complete response after neoadjuvant chemotherapy in breast cancer patients. *Breast*.

[25] Hirahara T., Arigami T., Yanagita S. (2019). Combined neutrophil-lymphocyte ratio and platelet-lymphocyte ratio predicts chemotherapy response and prognosis in patients with advanced gastric cancer. *BMC Cancer*.

[26] Diem S., Schmid S., Krapf M. (2017). Neutrophil-to-lymphocyte ratio (NLR) and platelet-to-lymphocyte ratio (PLR) as prognostic markers in patients with non-small cell lung cancer (NSCLC) treated with nivolumab. *Lung Cancer*.

[27] Mohsen A., Taalab M., Abousamra N., Mabed M. (2020). Prognostic significance of absolute lymphocyte count, absolute monocyte count, and absolute lymphocyte count to absolute monocyte count ratio in follicular non-Hodgkin lymphoma. *Clinical Lymphoma, Myeloma & Leukemia*.

[28] Tang Y., Hu H. Q., Tang F. X. (2020). Combined preoperative LMR and CA125 for prognostic assessment of ovarian cancer. *Journal of Cancer*.

[29] Trinh H., Dzul S. P., Hyder J. (2020). Prognostic value of changes in neutrophil-to-lymphocyte ratio (NLR), platelet-to-lymphocyte ratio (PLR) and lymphocyte-to-monocyte ratio (LMR) for patients with cervical cancer undergoing definitive chemoradiotherapy (dCRT). *Clinica Chimica Acta*.

[30] Wang Y., Huang G., Li Z. (2020). Prognostic significance of inflammatory biomarkers in patients with breast cancer skeletal metastases. *Cancer Management and Research*.

[31] Zhou W., Kuang T., Han X. (2020). Prognostic role of lymphocyte-to-monocyte ratio in pancreatic neuroendocrine neoplasms. *Endocrine Connections*.

[32] Xu W., Wu X., Wang X. (2020). Prognostic significance of the preoperative lymphocyte to monocyte ratio in patients with gallbladder carcinoma. *Cancer Management and Research*.

[33] Mandaliya H., Jones M., Oldmeadow C., Nordman I. I. (2019). Prognostic biomarkers in stage IV non-small cell lung cancer (NSCLC): neutrophil to lymphocyte ratio (NLR), lymphocyte to monocyte ratio (LMR), platelet to lymphocyte ratio (PLR) and advanced lung cancer inflammation index (ALI). *Translational Lung Cancer Research*.

[34] Forner A., Llovet J. M., Bruix J. (2012). Hepatocellular carcinoma. *The Lancet*.

[35] Schobert I. T., Savic L. J., Chapiro J. (2020). Neutrophil-to-lymphocyte and platelet-to-lymphocyte ratios as predictors of tumor response in hepatocellular carcinoma after DEB-TACE. *European Radiology*.

[36] Wang D., Bai N., Hu X. (2019). Preoperative inflammatory markers of NLR and PLR as indicators of poor prognosis in resectable HCC. *PeerJ*.

[37] Hanahan D., Weinberg R. A. (2011). Hallmarks of cancer: the next generation. *Cell*.

[38] Diakos C. I., Charles K. A., McMillan D. C., Clarke S. J. (2014). Cancer-related inflammation and treatment effectiveness. *The Lancet Oncology*.

[39] Chen L., Zhang Q., Chang W., Du Y., Zhang H., Cao G. (2012). Viral and host inflammation-related factors that can predict the prognosis of hepatocellular carcinoma. *European Journal of Cancer*.

[40] Ringelhan M., Pfister D., O'Connor T., Pikarsky E., Heikenwalder M. (2018). The immunology of hepatocellular carcinoma. *Nature Immunology*.

[41] Kurebayashi Y., Ojima H., Tsujikawa H. (2018). Landscape of immune microenvironment in hepatocellular carcinoma and its additional impact on histological and molecular classification. *Hepatology*.

[42] El-Serag H. B., Rudolph K. L. (2007). Hepatocellular carcinoma: epidemiology and molecular carcinogenesis. *Gastroenterology*.

[43] Dharmapuri S., Özbek U., Lin J. Y. (2020). Predictive value of neutrophil to lymphocyte ratio and platelet to lymphocyte ratio in advanced hepatocellular carcinoma patients treated with anti–PD-1 therapy. *Cancer Medicine*.

