# Supplementary material for: Blood Biomarkers Predict Survival Outcomes in Patients with Hepatitis B Virus-Induced Hepatocellular Carcinoma Treated with PD-1 Inhibitors
Source: J Immunol Res. 2022 Aug 17;2022:3781109. doi: 10.1155/2022/3781109 (PMC9402369; doi:10.1155/2022/3781109)
Supplement: Supplementary Materials — Supplementary Table 1: multivariate Cox proportional hazards regression models for progression-free survival. Supplementary Table 2: multivariate Cox proportional hazards regression models for progression-free survival. Supplementary Table 3: multivariate Cox proportional hazards regression models for overall survival. Supplementary Table 4: Wilcoxon signed-rank test of blood markers before and after treatment. [file 3781109.f1.docx]

Supplementary table 1. Multivariate Cox proportional hazards regression models for progression-free survival

|  | Multivariate analysis | | |  | |
| --- | --- | --- | --- | --- | --- |
| Variable | HR | 95% CI | p-value | |  |
| Child-Pugh Class  B vs A | 0.772 | 0.165-3.155 | 0.665 | |  |
| CNLC Stage  IIIb vs IIb and IIIa | 4.496 | 0.952-21.226 | 0.058 | |  |
| AFP  ≥ 400 vs< 400 ng/ml | 1.977 | 0.680-5.747 | 0.210 | |  |
| PVTT  Yes vs None | 9.311 | 1.178-73.626 | **0.034** | |  |
| NLR  ≥5 vs <5 | 2.909 | 0.950-8.905 | 0.061 | |  |
| Abbreviations: CNLC, China liver cancer staging; PVTT**:** portal vein tumor thrombus**;** NLR, neutrophil-to-lymphocyte ratio; AFP, α-fetoprotein. | | | | |  |

Supplementary table 2. Multivariate Cox proportional hazards regression models for progression-free survival

|  | Multivariate analysis | | |  | |
| --- | --- | --- | --- | --- | --- |
| Variable | HR | 95% CI | p-value | |  |
| Child-Pugh Class  B vs A | 0.546 | 0.107-2.794 | 0.468 | |  |
| CNLC Stage  IIIb vs IIb and IIIa | 3.563 | 0.756-16.782 | 0.108 | |  |
| AFP  ≥ 400 vs< 400 ng/ml | 1.621 | 0.566-4.636 | 0.368 | |  |
| PVTT  Yes vs None | 10.562 | 1.362-81.932 | **0.024** | |  |
| LMR  ≥1.8 vs <1.8 | 0.364 | 0.125-1.057 | 0.063 | |  |
| Abbreviations: CNLC, China liver cancer staging; PVTT**:** portal vein tumor thrombus**;** LMR, Lymphocyte-to-monocyte ratio; AFP, α-fetoprotein. | | | | |  |

Supplementary table 3. Multivariate Cox proportional hazards regression models for overall survival

|  | Multivariate analysis | | |  | |
| --- | --- | --- | --- | --- | --- |
| Variable | HR | 95% CI | p-value | |  |
| Child-Pugh Class  B vs A | 0.876 | 0.212-3.615 | 0.855 | |  |
| CNLC Stage  IIIb vs IIb and IIIa | 3.188 | 0.678-14.989 | 0.142 | |  |
| AFP  ≥ 400 vs< 400 ng/ml | 1.296 | 0.452-3.718 | 0.630 | |  |
| PVTT  Yes vs None | 10.358 | 1.318-81.425 | **0.026** | |  |
| LMR  ≥1.8 vs <1.8 | 0.607 | 0.206-1.789 | 0.365 | |  |
| Abbreviations: CNLC: China liver cancer staging; PVTT**:** portal vein tumor thrombus**;** LMR: Lymphocyte-to-monocyte ratio; AFP: α-fetoprotein. | | | | |  |

Supplementary Table 4. Wilcoxon sign rank test of blood markers before and after treatment

|  | Wilcoxon sign rank test | | |
| --- | --- | --- | --- |
| Variable | Median | Z | p-value |
| SII |  | -3.765 | **<0.0001** |
| pre-treatment | 482.74 |  |  |
| Post-treatment | 358.54 |  |  |
| PLR |  | -1.633 | 0.103 |
| pre-treatment | 124.51 |  |  |
| Post-treatment | 110.70 |  |  |
| NLR |  | -3.488 | **<0.0001** |
| pre-treatment | 3.30 |  |  |
| Post-treatment | 2.61 |  |  |
| LMR |  |  |  |
| pre-treatment | 2.44 | -1.979 | **0.048** |
| Post-treatment | 2.49 |  |  |
